# Dysfunction of the Left Dorsolateral Prefrontal Cortex is Primarily Responsible for Impaired Attentional Processing in Schizophrenia

**DOI:** 10.4306/pi.2008.5.1.52

**Published:** 2008-03-31

**Authors:** Jee Wook Choi, Bum Seok Jeong, Ji-Woong Kim

**Affiliations:** 1 Department of Psychiatry, Catholic University of Korea, Daejeon St. Mary's Hospital, Daejeon, Korea.; 2 Department of Psychiatry, Eulji University School of Medicine, Daejeon, Korea.; 3 Department of Psychiatry, Konyang University College of Medicine, Daejeon, Korea.

**Keywords:** Schizophrenia, Attention, Left dorsolateral prefrontal cortex, Magnetic resonance imaging

## Abstract

**Objective:**

The results for finding the deficit in the anterior cingulate (ACC) in schizophrenic patients (SZ) have been inconsistent according to the studies that used different Stroop tasks, which is unlike the deficit in the dorsolateral prefrontal cortex (DLPFC). In order to explore for the core region that's responsible for the selective attention deficit in SZ, we examined the results of a functional neuroimaging study, which involved the performance of the Stroop task using high or low prefrontal cortex related loads in SZ.

**Methods:**

Ten schizophrenic patients and healthy controls (HC) received functional magnetic resonance imaging (fMRI) during a Short/Long-term latency Stroop task. The changes in the neural activity were determined in well-known Stroop related regions of interest (ROIs) that consisted of the DLPFC, ACC, the parietal lobule and in the whole brain regions for both the main and interaction effects of latency, and the results of the short-term and long-term latency Stroop conditions were compared.

**Results:**

The response times for both the congruency and latency effects were more prolonged in the schizophrenics than in the HC. For the congruency effect, the schizophrenics showed significantly less activation in the same site of the left DLPFC in both the short-term and long-term latency conditions, as compared with the HC. For the latency effect, the regions of the left-side language network were over- or under-activated in the schizophrenics, as compared with the HC. Any interaction effect was not found for both the behavioral and fMRI results.

**Conclusion:**

Our results indicate that the deficit in the left DLPFC is the core impairment of attentional processing in schizophrenics, regardless of other possible interactions such as the latency effect.

## Introduction

The color-word Stroop task is a widely used measure of selective attention and executive functioning.^1^ Disproportionate slowing in the incongruent condition^1^,^2^ has been reported during performance of the Stroop task in those individuals diagnosed with schizophrenia. Functional neuroimaging studies have reported less Stroop-related activation in the anterior cingulate cortex (ACC)^3^ and the dorsolateral prefrontal cortex (DLPFC). Further, during the Stroop task, a positive correlation between the error rate and the activity in the ACC^4^ was noted in schizophrenics. Thus, previous studies have provided convincing evidence that dysfunction in the ACC may be related to attention problems, and these are prominent in schizophrenics.^5^

While DLPFC deficits have been consistently reported in schizophrenics, few studies have examined the relationship between the reduced speed in the DLPFC and the ACC during Stroop-related tasks. This is not surprising as the DLPFC and the ACC have different roles.^6^ Specifically, the ACC is involved in conflict monitoring, while the DLPFC plays a role in strategic processing.^6^ The activity of the ACC can be affected by the prefrontal cortex-related strategic load or a subject's prefrontal capacity, as well as the conflict load of the task. Further, ACC activity can be increased during conditions involving less prefrontal cortexrelated strategic processing or with greater ACC-related conflict engagement.^7^ The recent literature has not produced a consensus on these processes in regard to schizophrenia, possibly because dysfunction of the DLPFC, the ACC or a combination of both might be involved. If so, one of the reasons for the inconsistency in the neuroimaging literature regarding the cognitive dysfunction in schizophrenics could be accounted for by the prefrontal cortex-related strategic load, which is associated with well-known prefrontal dysfunction,^8^ or another cognitive load of an experimental task rather than with deficits in the ACC itself in schizophrenics. In fact, the level of the language-related cognitive load differs between the congruent and incongruent conditions in the color-word Stroop task. Moreover, language disturbances have been reported to be central to what is identified as schizophrenia.^9^,^10^ Therefore, the language-related load is a possible cognitive process that could affect the Stroop task.

To test our hypothesis, a Stroop task with short-term and long-term latency conditions was developed; as compared with traditional color-word Stroop tasks, this task has greater strategic and/or language loads, but it still maintains the same central conflict. In the short-term latency condition, the subjects were given less time to prepare for an upcoming Stroop task compared to the long-latency condition. To solve the subsequent Stroop task, the subjects should have used their strategic load-related prefrontal capacity, their language capacity or both. Using event-related functional magnetic resonance imaging (fMRI) of the short- and long-term latency Stroop effects, we examined the changes in the activity of the DLPFC, ACC and other language-related brain regions in schizophrenics, and schizophrenics have been reported to have failures in activating the lateral prefrontal cortex,^8^ and also language disturbance.^9^,^10^ We compared this data with that from the healthy control subjects.

## Methods

### Participants

The patient group consisted of 10 ubjects with a Diagnostic and Statistical Manual of Mental Disorders IV (DSM-IV)^11^ diagnosis of schizophrenia (Table 1). The patients were selected for the study if they were clinically stable (a Brief Psychiatric Rating Scale score less than 30) during the last 2 months and they were receiving stable doses of atypical antipsychotics. Seven schizophrenics were receiving olanzapine (mean dose=9.2±3.4 mg/day) and the remainder were receiving risperidone (mean dose=3.0±1.4 mg/day) at the time of the fMRI examination. Three patients were also receiving an antiparkinsonian drug and one patient was receiving benzodiazepine. The schizophrenics had thierpsychopathology measured with using the Positive and Negative Syndrome Scale^12^ (total score: 58.4±11.4; positive score 13.2±2.6; negative score 15.9±2.9). Ten healthy volunteers with no history of neurological disease, psychiatric disease, or drug/alcohol abuse were recruited as controls. All the subjects were strongly right-handed as assessed by the modified Edinburgh Handedness Inventory,^13^ and they had normal or corrected-to-normal vision and were native Korean speakers. The schizophrenic and control groups did not differ either for their age, the duration of their education or the duration of their parents' education. The study protocol was approved by the Ethical Committee, and all the subjects gave written informed consent for participation in this study.

### Short/long-term latency stroop

We developed the short & long-term latency Stroop test. For this task, a single trial included a cue, a latency period, a probe and an intertrial interval (ITI). A red, blue or yellow colored diagram of a T-shirt was presented as a cue, followed by a colored word as a probe after 3.5 or 7 seconds. Both the cue and the probe were presented for 0.5 seconds. The ITIs, i.e., the duration from the previous probe to the next cue, were 4 seconds long. The subjects had to press a button in order to indicate whether the color of the word was consistent with that of the T-shirt presented 3.5 or 7 seconds earlier. If both were the same color, then the subjects were required to press the left button with their dominant index finger (YES-response). If not, they were required to press the right one with their dominant middle finger (NO-response). The trial of 3.5 seconds for the latency period was called the 'Short-term latency' condition (probes of the short-term congruent or incongruent conditions: SCp or SIp; cues: SCc or SIc) and 7 seconds one was the 'long-term latency' condition (probes of long-term congruent or incongruent conditions: LCp or LIp; cues: LCc or LIc). The interval of 3.5 seconds in the 'Short-term latency' condition was decided on based on the working memory load with an interval of 3.5 seconds in the one-back condition, which was not different between the schizophrenics and the healthy subjects.^14^ This event-related fMRI paradigm using a button press response mechanism was created using OptSeq version 1.1 to optimize the sequence.^15^ Thus, all the trials were presented pseudorandomly in terms of both the congruency and latency effects, which they have. During the 410 seconds duration of the short/long-term latency Stroop, the trials were presented pseudorandomly in terms of congruency and latency. In the congruent trial, the probe consisted of one of the color words "RED", "BLUE" or "GREEN" printed in the congruent color. In the incongruent trial, the color word was printed in an incongruent color (e.g., "BLUE" printed in red) to produce incongruence between the color of the word and the name of the color. When a cue was presented, the subjects were not provided any information about the following probe. Thus, the subjects were not able to predict whether the following probe would be congruent or incongruent. Each condition involved the same number of YES and NO answers. The stimuli were presented to the subjects using an liquid crystal display (LCD) projector, and the images were back-projected onto a screen at the subjects' feet. Motor responses were made using a two-button mouse and they were recorded in real-time.

### Image preprocessing

The images were obtained with a General Electric Signa 1.5-T high-speed imaging system (modified by Advanced NMR Systems, Wilmington, MA). One hundred sixty four whole brain images of the blood oxygen level-dependent signal intensity were collected during the performance of the modified Stroop task with using a T2^*^-weighted echoplanar imaging (EPI) sequence (24 axial slices, 5 mm thickness, TR=2,500 ms, TE=50 ms, flip angle=90°). Image preprocessing and voxel-wise analyses were conducted using SPM5 (the Welcome Department of Cognitive Neurology, London, UK). The EPIs were normalized to a common mean and then movement-corrected with using a six-parameter rigid body translation. Each subjects' structural images were then coregistered to a common reference using a 12-parameter algorithm and they were smoothed using a three-dimensional Gaussian filter (8-mm full width at half maximum) to accommodate between-subject differences of the brain anatomy.

### Statistical analysis

#### Behavioral Data Analysis

The percentages of errors of each condition were entered into repeated-measures one-way analyses of variances (ANOVAs). Response times for the correctly answered probes were entered into repeated-measures ANOVAs with the subjects as a random effect (collapsing over items). Statistical significance was set at two tailed p values <0.05.

#### Individual functional Magnetic Resonance Imaging Analysis

Analysis on an individual basis was performed using the General Linear model with a design matrix that included 8 derivatives for the 4 cues conditions presented before 3.5 sec (short: SCc, SIc) or 7 sec (long: LCc, LIc) of the congruent and incongruent probes and the 2 congruent conditions (SCp, LCp) and the 2 incongruent (SIp, LIp) conditions in each short and long-latency condition. The analysis for the Stroop effect created a contrast image that held signal change values at each voxel for the incongruent stimuli compared with the congruent stimuli with both SIp-SCp of contrast in the short-term latency condition and both LIp-LCp of contrast in the long-term latency condition. The analysis for the latency effect made a contrast image that held signal change values at each voxel for the stimuli with short-term latency compared with long-term latency with both SCp-LCp of contrast in the congruent condition and both SIp-LIp of contrast in the incongruent condition.

Based on the most frequently reported activation sites in the literature,^16^ the analyses for the Stroop effect were conducted within priori regions of interest (ROI) that were limited to Brodmann's areas (BA) 24 and 32 in the medial prefrontal cortex, including the ACC and BA 8, 9 and 46 in the DLPFC, and the bilateral superior and inferior parietal lobules, with employing a mask created with the WFU Pick Atlas toolbox.^17^ The analyses for the latency effect were conducted across the whole brain voxels.

#### Group functional Magnetic Resonance Imaging Analysis

To make inferences at a single-group level, the blood oxygen level-dependent (BOLD) activities within the Stroop related ROIs for determining the Stroop effect and in the whole brain for determining the latency effect were analyzed with one-sample *t*-tests on a voxel-by-voxel basis in each of the subjects in the schizophrenic and healthy control groups. Both the group×congruency interactions and the group×latency interactions at each voxel were identified by constructing statistical maps showing the BOLD activity that significantly differed between the schizophrenics and the healthy controls on two-sample *t*-tests. The resulting t-values were transformed into Z-scores. A significance level of p<0.001 or p<0.005 (uncorrected for multiple comparisons) and the 10-voxels three-dimensional contiguity were used as the thresholds for the statistical maps. The anatomical localization of suprathreshold activity was determined by overlaying the activation maps onto the standard space image with using SPM5.

## Results

### Behavioral data (Table 2 and 3)

The ANOVAs for accuracy did not show a significant main effect for congruency, latency, the group or any interaction across the conditions. In both group, the Stroop effect was demonstrated by a longer response time at the incongruent condition than at the congruent condition, which was reflected by the significant main effect of congruency. The schizophrenics showed a greater Stoop effect (a longer response time) than the healthy control did, which was reflected in the congruency by the group interaction. The schizophrenics showed a latency effect (a longer response time) with a trend of significance in the short-latency condition than in the long-latency condition, which was unlike the healthy controls, as was reflected by the significant main effect of latency in the incongruent condition (p<0.07) and for the group by the latency interaction (p<0.052). However, the increase of the Stroop effect according to latency was not different between the groups, as was reflected by the lack of significant latency according to congruency by the group interaction.

### Functional magnetic resonance imaging data

#### The Main Effect of Both Stroop and Latency (Table 4)

For the main Stroop effect, both groups showed activation in both the left DLPFC and anterior ACC for both short-term and long-term latency. For the main effect of latency (short-latency condition>long-latency condition), the healthy controls showed activation in the left inferior frontal gyrus (IFG) in the congruency condition and the schizophrenics showed activation in the left inferior parietal lobule in the incongruent condition. For the effect of congruency by latency interaction, the left IFG was activated in both diagnostic groups, but activation of the right ACC was shown in the healthy controls.

#### Group Comparison of the Blood Oxygen Level-Dependent Activity Between the Schizophrenic and Control Groups (Table 5, Figure 1)

For the main Stroop effect, significantly less activation in the left DLPFC (BA 8, 9) in the schizophrenics, compared with the healthy controls, was demonstrated in both the short-term (red color dots) and long-term (green color dots) latency conditions. For the main effect of latency, significantly more activation was showed in the left superior temporal gyrus (STG) and in the left supramarginal gyrus in the schizophrenics and in left IFG in the healthy controls in the incongruent condition, but not in the congruent condition. However, there was not a significant effect of the group by congruency according to the latency interaction.

## Discussion

**FIGURE 1:**
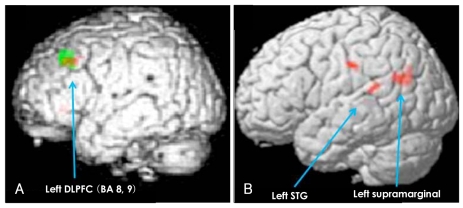
The results of comparing the groups. A: The stroop effect in both the short and long latency conditions: there are more activated sites in the left dorsolateral prefrontal cortex (DLPFC) of the healthy controls, compared with schizophrenics, in both the short-term (red colored dots) and long-(green colored dots) term latency conditions. B: The latency effect in both the congruent and incongruent conditions: there is more short latency-related activation in the left supramarginal gyrus and the left superior temporal gyrus (STG) of the schizophrenics, compared with the healthy controls, in the incongruent condition, but not in the congruent condition.

Our results demonstrated that for the Stroop effect, schizophrenics, as compared with the controls, showed poorer performance for the response time, and not for accuracy, which is in agreement with a previous study,^2^ and the deficits were in the left DLPFC, and not in the ACC, for both the short-term and long-term latency Stroop conditions. The deficits in the left DLPFC could be associated with the working memory^8^,^18^ or the continuous performance task deficits^19^ of the schizophrenics as our task paradigm had a time latency between the cue and the probe. However, our study showed that the deficits in the left DLPFC of the schizophrenics were not different between the shortterm and long-term latency Stroop conditions (Figure 1). Thus, deficits in the left DLPFC in schizophrenics might be related with the Stroop effect and it is due to neither the working memory nor the continuous performance load. The activation of the ACC did not show any difference between the two groups even though it did in each group analysis. Unlike the traditional Stroop test, our subjects were provided with 3.5 or 7 seconds to set a strategy for the following probe in the paradigm of the present study. Thus, the activity of the ACC for conflict monitoring may not be important to respond to the following prob.

For the latency effect (short>long latency condition), the schizophrenics, as compared with the controls, showed a longer response time, with a trend towards a level of significance, and dysfunction of the language network, which was both more activated in the left STG and the left supramarginal gyrus, and less activation in the left IFG (with a tendency towards a level of significance) in the incongruent condition. The behavioral latency effects might not have been due to deficits in the working memory or continuous performance in schizophrenics because the response time was more prolonged in the short-latency condition than in the long-latency condition. In the experimental design of the present study, the subjects might have been given less time to prepare for the upcoming Stroop task in the short-latency condition rather than in the long-latency condition. To prepare for the upcoming task, the subjects would require either a strategic or language process, or both. The significant congruency according to the latency (congruency by latency) effect (Table 3) suggested that the subjects would be given more time to prepare the strategy or to use other cognition, and especially language ability, to perform the Stroop task in the long-latency condition. A previous study indicated that the strategic process in the Stroop task was related with the DLPFC.^6^ Thus, the group difference for the latency effect might be related with the language network, which participated in encoding and recognizing the language component of the T-shirt color. The left IFG has been implicated in the language process, which is a role that's enabled by direct connections from the left IFG to the inferior and lateral temporal regions.^20^-^22^ fMRI studies have reported on the disruption of the functional connection in schizophrenics.^23^,^24^ One fMRI study found the functional deficit in the left IFG, along with overactivation of the superior temporal gyrus during the semantic process of schizophrenic patients.^23^ The other study that used a lexical-decision semantic priming paradigm demonstrated a less robust pattern of the inverse relationship between the semantic connectivity of words and activation in both the left IFG and the left temporal region in schizophrenics.^24^ Our fMRI group difference for the latency effect was consistent with the prior study on semantic processing. Thus, the activation discrepancy among the different brain regions indicates functional disruption within the left-side language network in schizophrenics. The latency effect was shown in the incongruent condition even though both the congruent and incongruent conditions had the same encoding load. Thus, more language load in the incongruent condition, compared with the congruent condition, may reveal the functional disruption of schizophrenia. Our schizophrenic group had a trend towards significance for the latency interaction [Table 3: p=0.052 (2×2×2 ANOVA), 0.08 (2×2 ANOVA) in the incongruent condition only] in the response time, and this was not related with the difference time periods between the cue and the probe, but it was related to the functional disruption of the left-side language network in the incongruent condition only. Yet it is interesting that both the behavioral and fMRI results showed no group difference in congruency according to the latency interaction (Table 3 and 5). This result suggests that Stroop-related DLPFC deficits might be independent of the functional disruption of the left-side language network.

The most possible confounding factor might have been a medication effect on cognition, and even for the second generation antipsychotics. However, dysfunction in the DLPFC and the language related regions has been reported in drug naive schizophrenics^25^,^26^ and in the nonaffected first relatives of schizophrenics.^27^ Further, a previous study reported increased frontal activity, including the DLPFC, during the Stroop task after treatment with second generation antipsychotics in schizophrenics.^28^ Also, the improvement of prefrontal brain function in the schizophrenics with using second generation antipsychotics was demonstarted electrophysologically as well as on neuropsychological testing for assessing the prefrontal brain function^29^ and this could reach the same level as the healthy controls.^30^ Thus, the Stroop-related DLPFC dysfunction might be mostly associated with a proper deficit of schizophrenics even though the effects of anticholinergic or antiparkinsonian drugs on cognition cannot be ignored.

There were some limitations given that this study involved a variety of diagnostic subtypes, the small sample size and the absence of matching between our patients and the healthy controls for variables such as IQ.

From all of these results, we concluded that the deficit in the left DLPFC is the core impairment of attentional processing in schizophrenics, regardless of other possible interactions such as the latency effect. The inconsistent behavioral results among the previous studies that used modified Stroop tasks^3^,^5^ would not due to the impairment of the attentional system, including the left DLPFC and the ACC. The different load from the experimental tasks on other cognitive systems, and especially the language systems, has to be considered in a schizophrenia study that uses a modified Stroop task. Further study using connectivity analysis such as dynamic causal modeling at the individual level may clarify the relationship between the DLPFC and other brain areas, including the ACC and the parietal lobule, for clarifying the core deficit region of the impaired attention in schizophrenics.

## Figures and Tables

**TABLE 1:**
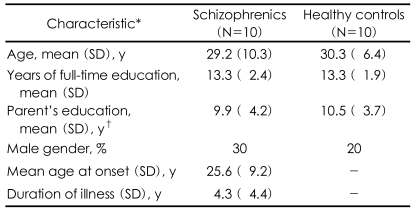
The sociodemographic and clinical characteristics of the schizophrenics and healthy controls

^*^Any differences in the demographic variables were not show between the patients and the control subjects, ^†^Averaged across the mother's and father's education, and neither of which significantly differed according to the group.

**TABLE 2:**
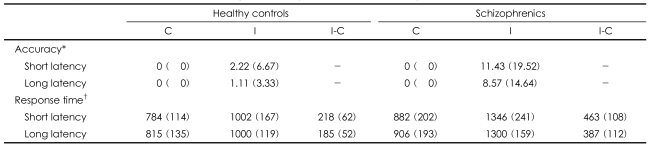
Response time in the short-term and long-term latency conditions for the healthy controls and schizophrenics

The behavioral data was acquired from 9 of 10 healthy controls and 8 of 10 schizophrenics because of the failure to record during the functional magnetic resonance (fMR) scanning. ^*^Mean percentage errors and the ^†^mean response times (in milliseconds), with standard deviations in parentheses, in each condition for the schizophrenics and healthy controls. The response times for each subject were averaged over all the correctly answered items. C: congruent condition, I: incongruent condition, I-C: di-fference in response time between the incongruent and congruent conditions, Short latency: short-term latency condition, Long latency: long-term latency condition

**TABLE 3:**
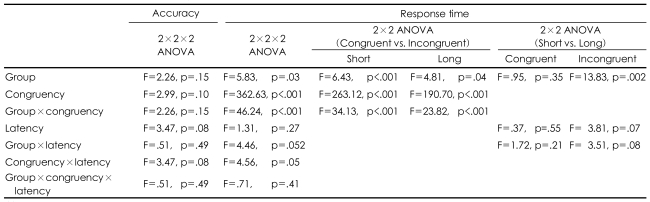
ANOVAs: behavioral accuracy and response times

ANOVA: analysis of variance, 2×2×2: group (healthy control vs. schizophrenics)×congruency (congruent vs. incongruent)×latency (short vs. long)

**TABLE 4:**
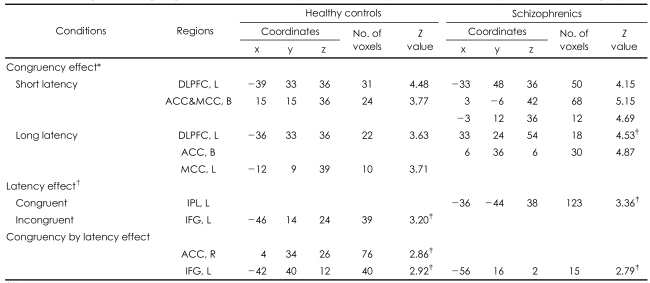
Brain regions showing a significant increase of activity for both the Stroop effect and the latency effect within each group

^*^Stroop effect: incongruent-congruent conditions, ^†^Latency effect: short-long latency conditions. Extent threshold of 10 voxels and height threshold with a p value 0.001 for the congruency effect (ROI analysis), except ^‡^in the long-term-latency Stroop effect in the schizophrenics with a p value of 0.005, ^‡^Statistical significance with a p value 0.005 for the latency effect and congruency by the latency interaction (whole brain analysis). DLPFC: dorsolateral prefrontal cortex, L: left, ACC: anterior cingulate cortex, MCC: middle cingulate cortex, B: bilateral, IPL: inferior parietal lobule, IFG: inferior frontal gyrus, R: right, ROI: regions of interest

**TABLE 5:**
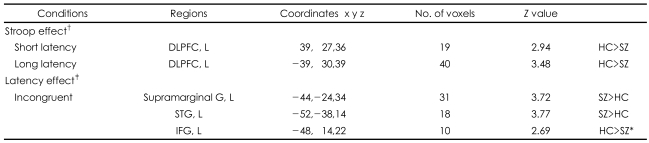
Brain regions showing a significant group difference both for the Stroop effect and the latency effect between groups

No region showed a group difference for the latency effect in the congruent condition. Extent threshold of 10 voxels and height threshold with a p value 0.005 (whole brain analysis). ^*^The left IFG showed a group difference in the incongruent condition for the latency effect with tendency towards significance (p<0.01). ^†^Stroop effect: incongruent-congruent conditions, ^‡^Latency effect: short-long latency conditions. DLPFC: dorsolateral prefrontal cortex, L: left, STG: superior temporal gyrus, IFG: inferior frontal gyrus.
